# Anti-Inflammatory Activity of Some Characteristic Constituents from the Vine Stems of *Spatholobus suberectus*

**DOI:** 10.3390/molecules24203750

**Published:** 2019-10-17

**Authors:** Xiao-Yan Liu, You-Bo Zhang, Xiu-Wei Yang, Yan-Fang Yang, Wei Xu, Wei Zhao, Kai-Feng Peng, Yun Gong, Ni-Fu Liu, Peng Zhang

**Affiliations:** 1 State Key Laboratory of Natural and Biomimetic Drugs, Department of Natural Medicines, School of Pharmaceutical Sciences, Peking University Health Science Center, Peking University, No. 38, Xueyuan Road, Haidian District, Beijing 100191, China; yanzi_89@163.com (X.-Y.L.); zybo5288@163.com (Y.-B.Z.); yangyanfang@bjmu.edu.cn (Y.-F.Y.); high-xu@163.com (W.X.); 2 Zhuzhou Qianjin Pharmaceutical Co., Ltd., Zhuzhou 412000, China; zw18273285046@163.com (W.Z.); kaikai81234@163.com (K.-F.P.); gongyun2002@126.com (Y.G.); 15873315432@163.com (N.-F.L.); pengzhangbjmu@163.com (P.Z.)

**Keywords:** *Spatholobus suberectus*, isoflavones, inhibition of nitric oxide production, anti-inflammatory activity, RAW264.7 macrophage cells

## Abstract

The dried vine stems of *Spatholobus suberectus* are commonly used in traditional Chinese medicine for treating gynecological and cardiovascular diseases. In this study, five new compounds named spasuberol A (**2**), homovanillyl-4-oxo-nonanoate (**5**), spasuberol C (**6**), spasuberoside A (**14**), and spasuberoside B (**15**), together with ten known compounds (**1**, **3**, **4**, **7**–**13**), were isolated from the dried vine stems of *S. suberectus*. Their chemical structures were analyzed using spectroscopic assays. This is the first study interpreting the detailed structural information of **4**. The anti-inflammatory activity of these compounds was evaluated by reducing nitric oxide overproduction in RAW264.7 macrophages stimulated by lipopolysaccharide. Compounds **1** and **8**–**10** showed strong inhibitory activity with half maximal inhibitory concentration (IC_50_) values of 5.69, 16.34, 16.87, and 6.78 μM, respectively, exhibiting higher activity than the positive drug l-*N*^6^-(1-iminoethyl)-lysine (l-NIL) with an IC_50_ value of 19.08 μM. The IC_50_ values of inhibitory activity of compounds **2** and **4**–**6** were 46.26, 40.05, 45.87, and 28.29 μM respectively, which were lower than l-NIL, but better than that of positive drug indomethacin with an IC_50_ value of 55.44 μM. Quantitative real-time polymerase chain reaction analysis revealed that assayed compounds with good anti-inflammatory activity, such as **1**, **6**, **9**, and **10** at different concentrations, can reduce the messenger RNA (mRNA) expression of some pro-inflammatory cytokines such as tumor necrosis factor α (TNF-α), nitric oxide synthase (iNOS), and cyclooxygenase 2 (COX-2). The anti-inflammatory activity and the possible mechanism of the compounds mentioned in this paper were studied preliminarily.

## 1. Introduction

*Spatholobi caulis* (SPC), belonging to family Leguminosae, is the dried vine stem of *Spatholobus suberectus* Dunn, chiefly distributed in Fujian Province and the Guangxi Zhuang autonomous region of China. SPC is an important ingredient of traditional Chinese medicine (TCM) recorded in the Pharmacopoeia of the People’s Republic of China [1], with demonstrated blood-activating and stasis-dissolving efficacy. In clinical practice, it is always used for treating rheumatic arthralgia, irregular menstruation, and paralysis in many Chinese formulas such as Jinkui Shenqi pills and Fukeqianjin formula (a classical prescription). Diverse pharmacological actions such as anti-inflammatory, anti-virus, hemagglutination, and lipid regulation were reported recently [2,3,4,5]. For chemical analysis, many active ingredients including flavonoids, isoflavones, and anthocyanins, with antioxidant activities [6,7], as well as the ability to treat renal injury [8] and adjust cholesterol metabolism in SPC [9], were isolated and identified, which inspired its further research.

Under the stimuli of mucosal injury, cellular damage, bacterial or viral infections, and other forms of cellular oxidative stress, different types of inflammatory responses can be caused, whereby pattern recognition receptors characterizing pathophysiological processes can be activated to identify the toxins infecting the host, such as lipopolysaccharide (LPS), lipoproteins, and lipoteichoic acids [10,11,12]. LPS is a surface antigen of the outer membrane of Gram-negative bacteria, through which macrophage cells are stimulated, thereby starting a cascade of reactions activating the release of nitric oxide (NO), prostaglandins, tumor necrosis factor α (TNF-α), pro-inflammatory cytokines interleukin 6 (IL-6) and cyclooxygenase 2 (COX-2), and other inflammatory mediators in the process of bacterial infection [13,14,15]. As an essential part of biochemical messengers involved with many physiological functions and immune responses, these inflammatory mediators play a complex role in inflammatory response; therefore, reducing inflammation by inhibiting the production of inflammatory mediators is a potential method of developing anti-inflammatory drugs. On the basis of the current struggle to discover more natural products as valuable resources for lead compounds for drug and pesticide design, the present study aims to identify more anti-inflammatory compounds in SPC, and further investigate the possible mechanism of NO inhibitory action of these isolated compounds using real-time quantitative polymerase chain reaction (qPCR).

## 2. Results and Discussion

### 2.1. Extraction and Isolation

The dried vine stems of *Spatholobus suberectus* (70 kg) were chopped and extracted with water (350 L twice, 2 h each) under reflux, and the combined extracts were concentrated at 75 °C in vacuum for 7 h to obtain a dry extractum (6 kg). The extractum was then suspended in water and segmented with ethyl acetate (EtOAc) and normal butanol (*n*-BuOH) to obtain the corresponding extract.

The EtOAc extract (270 g) was separated into seven fractions (Fr.1–Fr.7) by open silica gel column chromatography (SGCC) and eluted sequentially with chloroform (CHCl_3_)-methanol (MeOH) (80:1, 50:1, 30:1, 20:1, 10:1, 5:1, and 1:1, *v*/*v*). Fr.1 (19.9 g) was again eluted with CHCl3-MeOH (60:1, 40:1, 20:1, 10:1, 5:1, and 1:1, *v*/*v*) through open SGCC to yield six fractions (Fr.1A–F). The crystalline part of Fr.1A was purified by recrystallization in MeOH to obtain compound **1** (20.2 mg), while the amorphous part was divided into seven fractions (Fr.1A1–7) eluted with CHCl3-MeOH (60:1, *v*/*v*). Fr.1A7 was separated by reversed-phase semi-preparative HPLC (RPSP-HPLC) and eluted with MeCN-H_2_O (36:64, *v*/*v*, 8 mL/min); at a retention time (t_R_) of 58.2 min, compound **2** was obtained with a quality of 19.8 mg. Fr.1C was subjected to SGCC to get three fractions (Fr.1C1–3) with CHCl3-MeOH (40:1, *v*/*v*) as the elution phase, and compound **3** was isolated from Fr.1C3 using MeCN-H_2_O (35:65, *v*/*v*, 8 mL/min) as the elution that flowed through RPSP-HPLC. Fr.1D was sectioned to yield three fractions (Fr.1D1–3) using RPSP-HPLC eluted with MeCN-H_2_O (2:3, 11:9, 4:1, *v*/*v*). Fr.1D2 was further separated and purified with MeCN-H_2_O (36:64, *v*/*v*, 8 mL/min) by RPSP-HPLC, and compounds **4** (16.5 mg, t_R_ 37.6 min) and **5** (5.6 mg, t_R_ 39.1 min) were obtained. Fr.1E yielded compound **6** (8.3 mg) and two subfractions (Fr.1E1–2) after being subjected to Sephadex LH-20 CC using MeOH as the elution. Fr.1E2 was further detected by thin-layer chromatography (TLC) before being sectioned using RPSP-HPLC with MeCN-H_2_O (36:64, *v*/*v*, 8 mL/min) as the mobile phase, from which compound **7** (11.4 mg, t_R_ 26.3 min) was afforded.

Fr.2 (3.98 g) was gradually eluted with CHCl3-MeOH (100:1, 50:1, 20:1, 10:1, and 5:1, *v*/*v*) on SGCC to achieve five fractions (Fr.2A–E). Compound **8** (37.6 mg, t_R_ 45.9 min) was obtained from Fr.2C using MeCN-H_2_O (22:78, *v*/*v*, 8 mL/min) as the mobile phase pumped through RPSP-HPLC. Fr.2D was further sectioned and purified with MeCN-H_2_O (27:73, *v*/*v*, 8 mL/min) using RPSP-HPLC to get compounds **9** (52.7 mg, t_R_ 48.7 min) and **10** (48.2 mg, t_R_ 70.2 min). Fr.4 (26.6 g) yielded five fractions (Fr.4A–E) after being subjected to SGCC with a gradient of CHCl3-MeOH (80:1, 60:1, 40:1, 20:1, and 10:1, *v*/*v*) as the elution. Fr.4A was segmented by RPSP-HPLC with MeCN-H_2_O (35:65, *v*/*v*, 15 mL/min) as the mobile phase, and then purified by Sephadex LH-20 CC using MeOH as the elution to afford compounds **11** (422 mg) and **12** (68 mg). Fr.4D was purified repeatedly with the elution of MeOH on Sephadex LH-20 CC, and compound **13** (31.3 mg) was afforded. Fr.5 (25 g) was submitted to RPSP-HPLC with MeCN-H_2_O (10:90, *v*/*v*, 15 mL/min) as the mobile phase to obtain three fractions (Fr.5A–C). Fr.5A was purified repetitively with MeOH on Sephadex LH-20 CC to yield compound **14** (7.7 mg). Fr.6 (26.6 g) yielded three fractions (Fr.6A–C) after being fractionated by RPSP-HPLC eluted with MeCN-H_2_O (10:90, *v*/*v*, 15 mL/min); the middle fraction was further separated by RPSP-HPLC, and compound **15** (38.2 mg, t_R_ 49.9 min) was obtained using MeCN-H_2_O (22:78, *v*/*v*, 8 mL/min).

Spasuberol A ((3*S*,4*R*)-7,2′-dihydroxy-8-methoxy-4′,5′-methylenedioxy-isoflavan-4-ol, **2**): white powder (MeCN-H_2_O); [α]^25^_D_–450.0 (c 0.2, MeOH); ultraviolet (UV) (MeOH) λ_max_ nm (log ε): 203 (4.38), 309 (3.45); infrared (IR) (KBr) ν_max_: 3397, 2929, 1612, 1500, 1473, 1458, 1144, 1056, 1032 cm^−1^; for ^1^H NMR and ^13^C NMR data, see Table 1; high-resolution (HR) electrospray ionization (ESI) MS *m*/*z* 331.0840 [M − H]^−^ (calculated for C_17_H_15_O_7_, 331.0823).

Spasuberol B ((3*S*,4*R*)-7,2′-dihydroxy-4′,5′-methylenedioxy-isoflavan-4-ol, **4**): white powder (MeCN-H_2_O); [α]^25^_D_–699.0 (c 0.2, MeOH); UV (MeOH) λ_max_ nm (log ε): 204 (4.72), 309 (3.75); IR (KBr) ν_max_: 3250, 2933, 1619, 1596, 1474, 1458, 1144, 1032 cm^−1^; for ^1^H NMR and ^13^C NMR data, see Table 1; HR-ESI-MS *m*/*z* 285.0762 [M − H_2_O + H]^+^ (calculated for C_16_H_13_O_5_, 285.0763).

Homovanillyl-4-oxo-nonanoate (**5**): yellow-brown transparent oil (MeCN-H_2_O); UV (MeOH) λ_max_ nm (log ε): 203 (4.64), 381 (3.67); IR (KBr) ν_max_: 3418, 2932, 2256, 2128, 1713, 1645, 1517, 826 cm^−1^; for ^1^H NMR and ^13^C NMR data, see Table 2; HR-ESI-MS *m*/*z* 321.1699 [M − H]^−^ (calculated for C_18_H_25_O_5_, 321.1702).

Spasuberol C (3-(2′,3′,4′-trimethoxyphenyl)-6,7-dihydroxycoumarin, **6**): yellow amorphous powder (MeOH); UV (MeOH) λ_max_ nm (log ε): 207 (4.39), 249 (3.66), 356 (3.93); IR (KBr) ν_max_: 3301, 2937, 1687, 1579, 1509, 1471, 1299 cm^−1^; for ^1^H NMR and ^13^C NMR data, see Table 2; HR-ESI-MS *m*/*z* 345.0972 [M + H]^+^ (calculated for C_18_H_17_O_7_, 345.0974).

Spasuberoside A ((3*S*,4*R*)-4,2′-dihydroxy-4′-methoxy-isoflavanol-7-glucopyranoside, **14**): white powder (MeOH); [α]^25^_D_–50.0 (c 0.47, MeOH); UV (MeOH) λ_max_ nm (log ε): 203 (4.35), 225 (3.51), 285 (3.29); IR (KBr) ν_max_: 3385, 3205, 2988, 2918, 1624, 1595, 1586, 1508, 1496 cm^−1^; for ^1^H NMR and ^13^C NMR data, see Table 3; HR-ESI-MS *m*/*z* 433.1499 [M − H_2_O + H]^+^ (calculated for C_22_H_25_O_9_, 433.1499).

Spasuberoside B ((3*R*)-vestitol-2′-*O*-β-d-glucopyranoside**, 15**): white powder (MeCN-H_2_O); [α]^25^_D_–150.0 (c 0.2, MeOH); UV (MeOH) λ_max_ nm (log ε): 203 (4.63), 224 (4.00), 283 (3.48); IR (KBr) ν_max_: 3335, 2916, 2255, 2127, 1707, 1615, 1509, 1459, 1162, 825 cm^−1^; for ^1^H NMR and ^13^C NMR data, see Table 3; HR-ESI-MS *m*/*z* 433.1488 [M − H]^−^ (calculated for C_22_H_25_O_9_, 433.1499).

### 2.2. ECD Calculation

A Monte Carlo simulation was used to analyze the configurations of compounds using the MMFF94 molecular mechanics force field. The TDDFT method at the B3LYP/6-31G(d) level was used to optimize the conformation, the stability of which was confirmed by calculating the frequencies at the same level. After that, the TDDFT method at the B3LYP/6+31G(d) level with the CPCM model in MeOH was applied to calculate the ECD stable conformers. Gaussian 09 was used to calculate all relevant parameters, and SpecDis v 1.51 was used to simulate the ECD spectra with a half spectral bandwidth of 0.3 eV. Moreover, the ECD spectra were gained according to the Boltzmann-calculated contribution of different conformers.

### 2.3. Structural Elucidation of Isolated Compounds **1**–**15**

**Figure 1:**
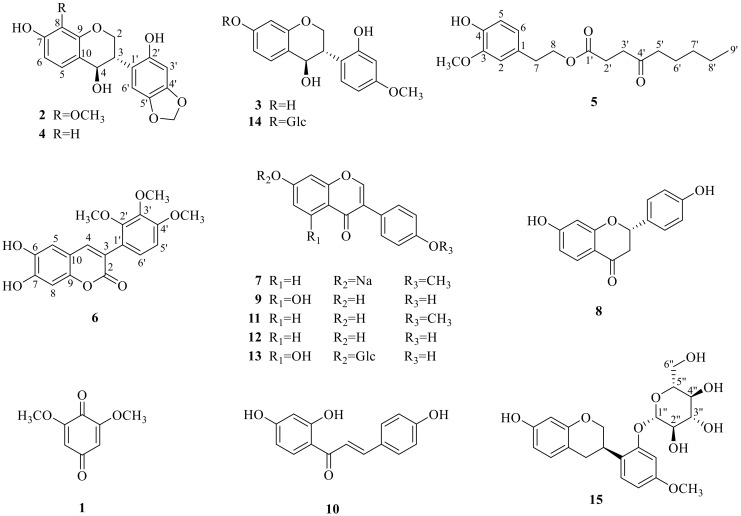
The structures of compounds **1**–**15** isolated from the vine stems of *Spatholobus suberectus.*

Compounds (**1**–**15**) including five new ones (**2**, **5**, **6**, **14**, **15**) afforded by the EtOAc and *n*-BuOH extractions were identified by spectroscopic analysis. The nine known compounds were further compared to the reported literature data and identified as 2,6-dimethoxy-1,4-benzoquinone (**1**) [16], 4,7,2′-trihydroxy-4′-methoxy-isoflavanol (**3**), liquiritigenin (**8**) [17], formononetin sodium (**7**) [18], genistein (**9**), formononetin (**11**), daidzein (**12**) [19], isoliquiritigenin (**10**) [20], and genistin (**13**) [21]. Their chemical structures are shown in Figure 1.

**Figure 2:**
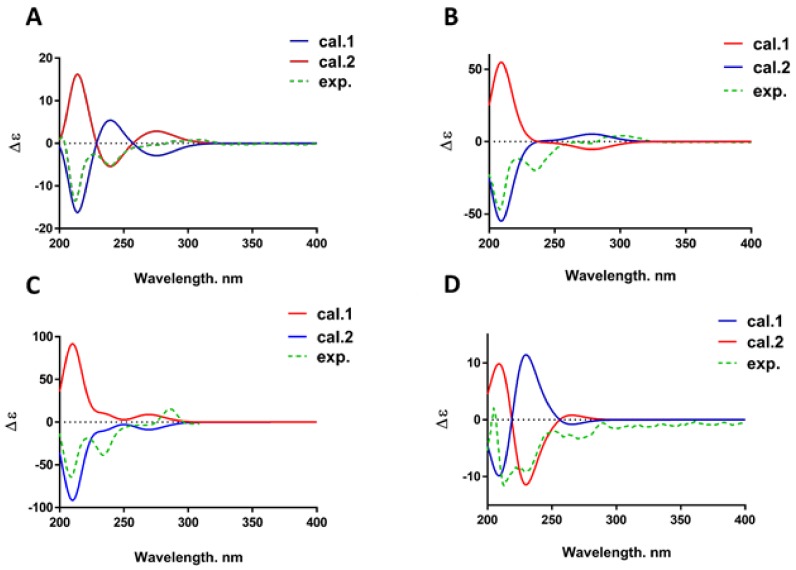
The ECD spectrum of compounds **2**, **4**, **14**, and **15** (**A**–**D**).

**Figure 3:**
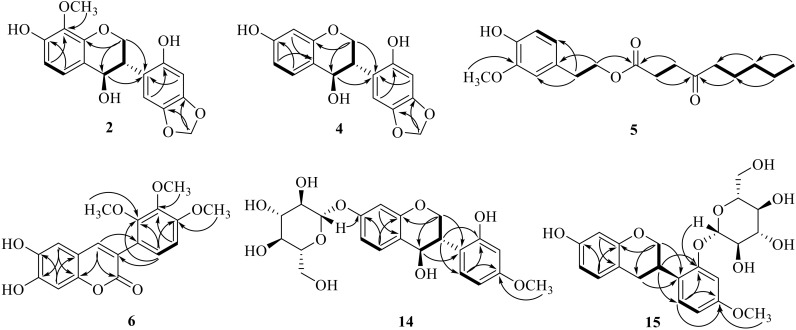
The ^1^H–^1^H COSY (**—**) and key HMBC (→ from H to C) correlations of compounds **2**, **4***–***6***,* **14**, and **15**.

Compound **2** was obtained as a white powder. HR-ESI-MS demonstrated a negative ion at *m*/*z* 331.0840 [M − H]^−^ (calculated for 331.0823), which corresponded to a molecular formula of C_17_H_16_O_7_. The IR spectrum of **2** showed maximum absorption bands due to a hydroxyl group (3397 cm^−1^) and aromatic ring (1612, 1458 cm^−1^). The ^1^H NMR and ^13^C NMR data (Table 1) of **2** were similar to those of pumilanol [22], an isoflavanol, except for the coupling pattern of the A-ring in the ^1^H NMR spectrum. Complete unambiguous assignments of the ^1^H and ^13^C NMR signals (Table 1) of **2** were made by combination of ^1^H–^1^H COSY, HSQC, HMBC, and DEPT spectra. An HMBC experiment showed a methoxyl group at δ_H_ 3.66 (3H, s) correlating with carbon at δ_C_ 135.8 (C-8), suggesting that this methoxyl group should be located at C-8. On this basis, the planar structure was identified as 7,2′-dihydroxy-8-methoxy-4′,5′-methylenedioxy-isoflavan-4-ol. Comparing to the reported data [23], a negative Cotton effect at 210 nm and a positive Cotton effect between 280 nm and 300 nm in the ECD spectrum revealed a 3*S*,4*R* absolute configuration in **2** (Figure 2A). The key correlations in the ^1^H–^1^H COSY and HMBC spectra of **2** are shown in Figure 3. It was a novel compound, given the trivial name spasuberol A.

Compound **4** was identified as a white powder with the molecular formula C_16_H_14_O_6_ according to the HR-ESI-MS ion of *m*/*z* 285.0762 [M − H_2_O + H]^+^ (calculated for 285.0763). The IR spectrum of **4** showed maximum absorption bands due to a hydroxyl group (3250 cm^−1^) and aromatic ring (1596, 1458 cm^−1^). All of the ^1^H NMR and ^13^C NMR data (Table 1) of **4** were similar to those of **2** except for the absence of a methoxyl group signal and an additional proton signal at δ 6.27 (1H, d, *J* = 2.4 Hz, H-8) on the mother nucleus. Thus, the planar structure was identified as 7,2′-dihydroxy-4′,5′-methylenedioxy-isoflavan-4-ol. Although it was reported in a previous study [24], this is the first study to interpret its detailed structural information. Its configuration was 3*S*,4*R* according to the CD and ECD spectra (Figure 2B). Based on the abovementioned structural data, the structure of **4** was identified as (3*S*,4*R*)-7,2′-dihydroxy-4′,5′-methylenedioxyisoflavan-4-ol and given the trivial name spasuberol B.

Compound **5** was a yellow-brown transparent oil. The molecular formula was C_18_H_26_O_5_ inferred from the HR-ESI-MS ion of *m*/*z* 321.1699 [M − H]^−^ (calculated for 321.1702). The low-field region of its ^1^H NMR (Table 2) indicated a benzene ring as part of this structure, of which the hydrogens on C-1, C-3, and C-4 were replaced by other substituent groups when conducting a further analysis combined with the ^13^C NMR experiment (Table 2). A hydroxyl group proton at δ_H_ 9.00 (1H, s), a methoxyl group at δ_H_ 3.74 (3H, s), two linked methylenes at δ_H_ 2.74 (*J* = 7.0 Hz) and δ_H_ 4.13 (*J* = 7.0 Hz), and amyl group signals at δ_H_ 0.85, 1.25, 1.45, and 2.39 in the high nuclear magnetic field were found in the ^1^H NMR spectrum. The ^13^C NMR spectrum revealed two carbonyl groups at δ 172.4 and 209.1, and two carbons signals at δ_C_ 147.6 (C-2) and δ_C_ 145.3 (C-3) of the benzene ring, revealing the connections with hydroxyl and methoxyl groups, respectively. The HMBC correlations (Figure 3) indicated that the methoxyl group was attached to C-3, in line with the data from δ_H_ 3.74 to δ_C_ 145.3 (C-3). The relevance of H-7 to C-1, C-2, and C-6 indicated that C-7 was linked to the C-1 position. Furthermore, the chemical shift value of C-8 at δ_C_ 65.0 was significantly higher than that of C-7, and the HMBC correlation of H-8 and C-1′ identified an oxygen atom between C-8 and C-1′. C-4′ was deemed a carbonyl carbon between C-3′ and C-5′ because of the lack of hydrogen on C-4′. It was a novel compound, given the name homovanillyl-4-oxo-nonanoate.

The exterior trait of novel compound **6** was a yellow amorphous powder. Its molecular formula, C_18_H_16_O_7_, was established by the HR-ESI-MS ion of *m*/*z* 345.0972 [M + H]^+^ (calculated for 345.0974). In the IR spectrum of **6**, the absorption band at 3301 cm^−1^ was caused by the hydroxyl group, whereas 1687 cm^−1^ represented the existence of α,β-unsaturated six-membered lactone; these corollaries were further supported by the presence of a carbon signal at δ_C_ 160.5 in the ^13^C NMR spectrum. Its NMR spectroscopic data had a slight difference in the position of the substituents compared with that of 6,7-dihydroxy-3-(3′,4′,5′-trimethoxyphenyl)chromenone [25], a coumarinoid compound. In the substituted group of C-3, the ^1^H NMR data showed a pair of *ortho*-aromatic protons at δ_H_ 6.64 (1H, d, *J* = 8.6 Hz) and δ_H_ 6.86 (1H, d, *J* = 8.6 Hz). In the HMBC experiment, the proton signal of δ_H_ 6.86 and δ_H_ 6.64 showed long-range correlation with the carbon signals of δ_C_ 121.4 (C-3) and δ_C_ 120.6 (C-1′), respectively. Therefore, the signal δ_H_ 6.86 was assigned to the H-6′ and the signal δ_H_ 6.64 was assigned to the H-5′. Based on extensive analysis of the HMBC spectrum, significant long-range correlations were observed between δ_H_ 6.86 and δ_C_ 151.8, as well as between δ_H_ 6.64 and δ_C_ 151.8, 140.8, and 120.6. Thereby, the signals of δ_C_ 151.8 and δ_C_ 140.8 belonged to C-4′ and C-3′. Based on comprehensive analysis of the HSQC and HMBC spectral data, methoxyl groups at δ_H_ 3.82 (3H, s) and δ_H_ 3.77 (3H, s) were linked to C-4′ and C-3′, respectively. Thus, the structure of **6** was determined as 3-(2′,3′,4′ -trimethoxyphenyl)-6,7-dihydroxycoumarin, named spasuberol C.

Compound **14** was obtained as a white powder with the molecular formula C_22_H_26_O_10_, speculated from the HR-ESI-MS ion of *m*/*z* 433.1499 [M − H_2_O + H]^+^ (calculated for 433.1499). The NMR spectra (Table 3) exhibited extra glucopyranosyl group signals in **14** when compared to the signals of 4,7,2′-trihydroxy-4′-methoxy-isoflavanol [17]. Its ^1^H–^1^H COSY correlations from H-6 at δ_H_ 3.74 to H-5 at δ_H_ 7.39 and H-8 at δ_H_ 6.56 indicated that the glucopyranosyl group was maybe located at C-7 (δc 158.6). Based on the HMBC correlation and HR-ESI-MS data, the relevance of H-1″ at δ_H_ 4.84 and C-7 further confirmed that C-1″ of the glucopyranosyl group was linked to C-7 via an oxygen bond. The negative and positive Cotton effects at 210 nm and around 290 nm, respectively, in the ECD spectrum implied a 3*S*,4*R* absolute configuration of **14** (Figure 2C). Finally, the structure of **14** was determined as (3*S*,4*R*)-4,2′-dihydroxy-4′-methoxyisoflavanol-7-glucopyranoside, named spasuberoside A.

Compound **15** was a white powder with the molecular formula C_22_H_26_O_9_ according to the HR-ESI-MS ion of *m*/*z* 433.1488 [M − H]^−^ (calculated for 433.1499). The NMR spectral data of **15** were similar to those of (3*R*)-vestitol-7-*O*-glucoside [26] except for the connective position signals of a glucopyranosyl group. A hydroxyl group at δ_H_ 9.13 and a methoxyl group at δ_H_ 3.72 were found in the ^1^H NMR spectrum. Correlations from H-6 at δ_H_ 6.29 to H-5 at δ_H_ 6.87, and from H-6′ at δ_H_ 7.07 to H-5′ at δ_H_ 6.55 based on ^1^H–^1^H COSY indicated two substituents on C-7 and C-4′, and a deeper estimation from HSQC and HMBC correlations showed that the hydroxyl and methoxyl groups were linked together with C-7 and C-4′, respectively. Furthermore, C-1″ of the glucopyranosyl group was linked to C-2′ via an oxyglycoside bond on the basis of the HMBC spectrum showing H-1″ (δ_H_ 4.84) correlated to C-2′ (δ_C_ 156.3). The CD and ECD data between 260 nm and 280 nm showed a negative Cotton effect (Figure 2D), which implied a 3*R* absolute configuration at C-3. Accordingly, the structure of **15** was deduced as (3R)-vestitol-2′-*O*-β-d-glucopyranoside, a new member of the isoflavan group, named spasuberoside B.

### 2.4. Activity of Inhibiting NO Overproduction

As a kind of pro-inflammatory mediator, NO plays a complex role and participates in many different kinds of inflammatory reactions [27,28], the level of which usually varies to different pathological degrees. The isolated novel compounds and 10 known ones in this research were chosen to evaluate the activity of inhibiting NO released by RAW264.7 cells after being stimulated by LPS.

All the assayed compounds showed varying inhibitory activities on NO production, and the half maximal inhibitory concentration (IC_50_) values are listed in Table 4. Compounds **1**, **6**–**11** had potent inhibition, whilst the other eight ones had moderate inhibition activities compared with the inducible NO synthase inhibitor, l-*N*^6^-(1-iminoethyl)-lysine (l-NIL). Better yet, compounds **1** and **10** showed a more significant inhibitory effect on NO overproduction than L-NIL (*p* < 0.01), The median cytotoxic concentration (CC_50_) and selectivity index (SI) values of compound **10** were 133.44 μM and 19.68, respectively, which implied a lower cytotoxicity and a higher selectivity than compound **1** (CC_50_ and SI values were 26.23 μM and 4.61, respectively). When compared with indomethacin (IND), another positive drug in the study of anti-inflammatory, antipyretic, and analgesic properties [29,30], compounds **1**, **8**–**10** displayed extremely significant effects on NO inhibition (*p* < 0.001), and the activities of compounds **7**, **11**, and **12** were also significantly better than that of IND (*p* < 0.01).

From Table 4, it is worth mentioning that the NO inhibitory activities of the selected aglycones were superior to the glycosides. Whether comparing compound **3** with **14** or **9** with **13**, the inhibitory activities decreased significantly due to the 7-hydroxyl group replaced by a glucosyl group, in accordance with a previous study [31]. In addition, the flavonoids with a 4-carbanyl group showed a stronger anti-inflammatory activity on NO overproduction than compounds without the 4-carbanyl group. Comparing compounds **7**, **11**, and **12**, the presence of sodium had little effect on activity; however, when the 4′-methoxyl group on ring B was substituted with a 4′-hydroxyl group, the inhibition activity also decreased relatively. Furthermore, seen from compounds **2** and **4**, the activities were barely affected by the presence of the 8-methoxyl group.

### 2.5. Analysis of Messenger RNA (mRNA) Expression Levels of Inflammatory Factors

**Figure 4:**
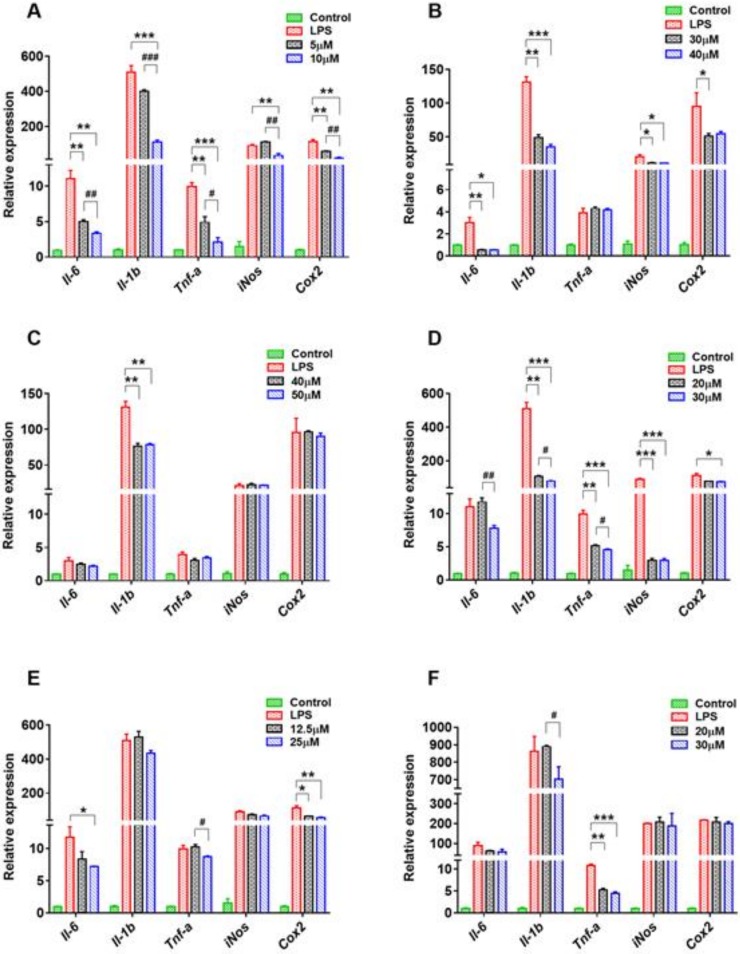
Effect of compounds **1**, **3**, **4**, and **6**–**8** (**A**–**F**) on the messenger RNA (mRNA) levels of interleukin-6 (IL-6), IL-1β, tumor necrosis factor α (TNF-α), inducible nitric oxide synthase (iNOS), and cyclooxygenase 2 (COX-2) in RAW264.7 cells stimulated by lipopolysaccharide (LPS). Data are presented as means ± standard error of the mean (SEM), *n* = 3; * *p* < 0.05, ** *p* < 0.01, *** *p* < 0.001, treatment vs. LPS model; ^#^ *p* < 0.05, ^##^ *p* < 0.01, ^###^ *p* < 0.001, high concentration vs. low concentration.

**Figure 5:**
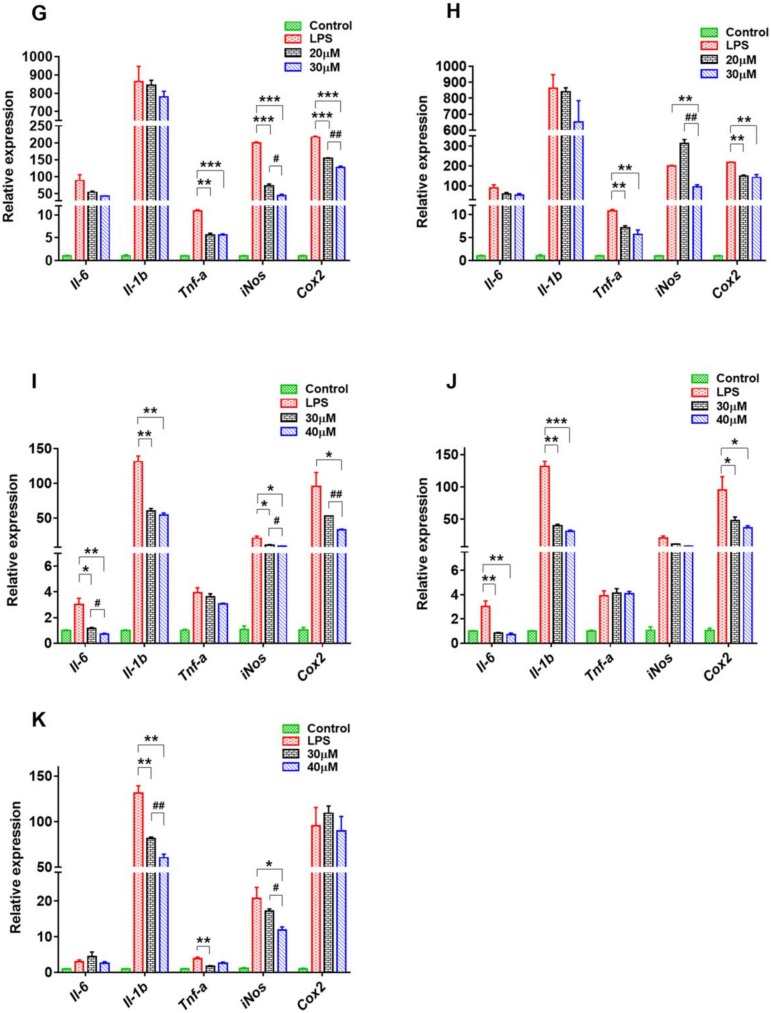
Effect of compounds **9**–**13** (**G**–**K**) on the mRNA levels of IL-6, IL-1β, TNF-α, iNOS, and COX-2 in RAW264.7 cells stimulated by LPS. Data are presented as means ± SEM, *n* = 3; * *p* < 0.05, ** *p* < 0.01, *** *p* < 0.001, treatment vs. LPS model; ^#^ *p* < 0.05, ^##^ *p* < 0.01, ^###^ *p* < 0.001, high concentration vs. low concentration.

From a growth perspective, underlying mechanisms should be determined to explain how these compounds from SPC resist or reduce inflammation via inhibiting the excessive production of NO. The effects of compounds **1**, **3**, **4** and **6**–**13** on messenger ribonucleic acid (mRNA) expression levels of IL-6, pro-inflammatory cytokines interleukin 1β (Il-1β), TNF-α, and proteases inducible nitric oxide synthase (iNOS), and COX-2 in RAW 264.7 cells were tested using qPCR (Figure 4; Figure 5). Some notable information from Figure 4; Figure 5 revealed that these compound concentrations dependently and markedly reduced the mRNA expression levels of the abovementioned cytokines and proteases, especially compound **1** at concentrations of 5 μM and 10 μM (Figure 4A). Compounds **3** and **4**, belonging to flavanols, could inhibit the mRNA expression of Il-1β at concentrations of 40 μM and 50 μM, suggesting a moderate inhibitory activity compared with the other compounds (Figure 4B,C). Compared with the LPS group, the mRNA expressions of IL-1β, TNF-α and iNOS were reduced significantly by compound **6** at concentrations of 20 μM (*p* < 0.01) and 30 μM (*p* < 0.001), which may be associated with the three methoxyl groups on this coumarin mother nucleus (Figure 4D).

Compounds **7**, **11**, and **12** influenced these mRNA expression levels to a certain degree (Figure 4E and Figure 5I,J). In addition, compound **11** also notably downregulated the mRNA levels of iNOS and COX-2, in addition to the inhibition of mRNA levels of IL-6 and IL-1β by compound **12**, confirming that the 4′-methoxyl group on ring B may be a critical factor for these isoflavones to exhibit anti-inflammatory activity. The hydroxyl group at C-7 was also closely associated with the activity of compound **7**. Once the proton was substituted by sodium, it seemed to negate the effect of compound **7** on these cytokines and proteases except for COX-2.

Compounds **8** and **10** are isomers with remarkable inhibitory function on NO overproduction, and both of them could significantly downregulate the mRNA expression of TNF-α (Figure 4F and Figure 5H). Furthermore, both the NO overproduction and the mRNA levels of iNOS and COX-2 were more obviously inhibited when ring C of compound **8** was opened. The mRNA levels of TNF-α, iNOS, and COX-2 in RAW264.7 cells were strikingly downregulated when treated with compound **9** at concentrations of 20 μM and 30 μM (Figure 5G). Compared with compound **9**, compound **13** with weaker inhibitory activity could not inhibit these three mRNA expressions (Figure 5K), but the mRNA level of IL-1β declined sharply, perhaps because of the existence of a glucose group at C-7.

The information motioned above indicated that either the satisfactory NO inhibitory activity or the regulation of mRNA expression of inflammatory factors was closely related to the chemical structures of these selected compounds. Compound **1** could be considered for structure modification to further reduce its cytotoxicity and increase its anti-inflammatory activity.

## 3. Experimental Section

### 3.1. General Experimental Procedures

The specific optical rotations were measured on Autopol III polarimeter (Rudolph Research Analytical, Flanders, NJ, USA). UV spectra were recorded using a Cary 300 UV–visible light (UV-Vis) spectrophotometer (Varian, Inc., Palo, Alto, CA, USA) with MeOH as the solvent, and IR spectra were determined by a Nexus 470 Fourier-transform IR (FT-IR) spectrometer (Thermo Nicolet, Inc., Madison, WI, USA) using the KBr squashed method. of the one- and two-dimensional (1D and 2D) NMR spectra were obtained from a Bruker AV 400 spectrometer (Bruker, Karlsruheb, Baden-Wuerttemberg, Germany), while tetramethylsilane (TMS) was used as the internal standard. High-resolution electron spray ionization mass spectrometry (HR-ESI-MS) was taken from a Waters Xevo G2 Q-TOF mass spectrometer (Waters, Milford, MA, USA).

Thin-layer chromatography (TLC) was carried out on silica gel GF_254_ plates (Merck, Darmstadt, Germany). Column chromatography (CC) was performed with silica gel (200–300 mesh, Qingdao Marine Chemical Factory, China). Sephadex LH-20 was purchased from Mitsubisi Petrochemical Co. (Mitsubisi Petrochemical Co., Tokyo, Japan). Compounds were isolated and further purified by a reversed-phase semi-preparative HPLC (RPSP-HPLC) system equipped with an LC3000 UV detector, a LabTech P600 pump, and a 7725i Rheodyne injector (Rheodyne, Cotati, CA, USA) with a loop of 5 mL, and a preparative Phenomenex Prodigy C_18_ column (250 × 21.2 mm inner diameter (i.d.), 10 µm; Phenomenex, Torrance, CA, USA) protected by a C_18_ guard column (8 × 4 mm i.d., 5 µm; Dikma, Beijing, China). A CXTH 3000 Chromsoftware workstation was used to maintain and test the operating state.

MeCN and MeOH (HPLC grade) were purchased from Fisher Scientific (Fair lawn, NJ, USA). MeOH, EtOAc, CHCl_3_, *n*-BuOH, isopropanol, and absolute alcohol (analytical grade) were purchased from Beijing Chemical Works (Beijing, China). Dulbecco’s modified Eagle’s medium (DMEM), fetal bovine serum (FBS), penicillin-streptomycin solution, and phosphate-buffered saline (PBS) were purchased from Gibco^TM^ (Life Technologies Inc., Grand Island, NY, USA). LPS, Griess reagent, 3-(4,5-dimethyl-2-thiazolyl)-2,5-diphenyl-2*H*-tetrazoliumbromide (MTT), L-NIL, and IND were obtained from Sigma Aldrich Co. (St. Louis, MO, USA). The RAW264.7 cells were supplied by the Cell Resource Center, IBMS, CAMS/PUMC (Beijing, China).

### 3.2. Plant Material

The dried vine stems of *Spatholobus suberectus* were harvested from Pu’er city, Yunnan Province, China (23°19′ north (N), 100°30′ east (E)) and kindly supplied by Zhuzhou Qianjin Pharmaceutical Co., Ltd. The species was identified by Prof. Xiu-Wei Yang at the School of Pharmaceutical Sciences, Peking University Health Science Center, Peking University. A voucher specimen (No. Y008-1608010) is stored at the State Key Laboratory of Natural and Biomimetic Drugs (Peking University, Beijing, China).

### 3.3. Biological Study

#### 3.3.1. Cytotoxicity Assay

The toxicities of different concentrations of compounds to cells were evaluated using the MTT assay [32,33]. Firstly, the RAW 264.7 cells were maintained in DMEM with 10% FBS at 37 °C in a humid atmosphere containing 5% CO_2_. Then, the cells were seeded in 96-well plates (3 × 10^4^ cells/well) after being passaged three times at a 1:3 split ratio. A sequence of concentrations of the tested compounds (3.12 μM–200 μM) with or without LPS (1 μg/mL) was added to stimulate cells for 24 h. After being cultured for 24 h, the supernatant (100 μL) was transferred out and MTT (5 mg/mL, 20 μL for each well) was added, and the mixed solvent of 0.012 M HCl, 5% i-PrOH, and 10% sodium dodecyl sulfate (100 μL) was added 4 h later. The absorbance value was measured at 492 nm by a Thermo Multiskan MK3 microplate reader (Thermo-Labsystem, Franklin, MA, USA).

#### 3.3.2. NO Inhibition Assay

The Griess method is generally used for determining NO concentration in anti-inflammatory experiments [34,35]. The supernatant removed from the 96-well plates mentioned in Section 3.3.1 was interacted with Griess reagent (100 μL), and the absorbance was measured at 540 nm after 15 min. Two drugs, l-NIL and IND, were used as positive controls for comparative analysis in the test.

#### 3.3.3. qPCR Assay

The RAW264.7 cells were seeded in 12-well plates at 3 × 10^4^ cells/well; after being stimulated by LPS (1.0 μg/mL) and treated with two different concentrations of compounds for 24 h, the total RNA was extracted from the cells with Trizol reagent (Invitrogen, Thermo Fisher Scientific Inc., Waltham, MA, USA). After measuring the concentration, 1 μg of total RNA was used to obtain complementary DNA (cDNA) with a qRT super mix (Bimake Co., Ltd., Houston, SD, USA). The amplification of cDNA was carried out on a Quantitative RT-PCR system (Agilent Technologies, Stratagene, Inc., Santa Clara, CA, USA) using SYBR green qPCR master mix (Bimake Co., Ltd., Houston, SD, USA). The thermal cycling conditions were as follows: 95 °C for 10 min, 40 cycles of 95 °C for 30 s, 55 °C for 1 min, and 72 °C for 1 min, followed by 95 °C for 1 min, 55 °C for 30 s, and 95 °C for 30 s. The sequences of the primers used in our experiment are listed in Table 5.

#### 3.3.4. Statistical Analysis

The IC_50_ values for NO inhibitory effects were calculated using SPSS v.20.0 (SPSS Inc., Chicago, IL, USA) and results were given as means ± SD. The mRNA expression levels were compared using variance analysis with GraphPad Prism 7.0 (GraphPad Software Inc., San Diego, CA, USA), and results were expressed as means ± SEM. All tests were carried out in triplicate.

## 4. Conclusions

In conclusion, five new and 10 known compounds were isolated and identified from the vine stem of *S. suberectus* in the current study. Despite there being no novel maternal nucleus skeleton found in these new compounds, the research still further enriched the compound library of SPC, and made a clear comparison with some known compounds sharing the same mother nucleus in terms of toxicity and activity. According to the results of inhibition of the overproduction of NO in Raw264.7 cells induced by LPS, compound **1**, 2,6-dimethoxy-1,4-benzoquinone, showed the best activity but the highest cytotoxicity compared to the other assayed compounds because of its structural particularity. Therefore, the aims of toxicity reduction and efficiency enhancement could be achieved by structural modification. In general, flavonoid aglycones have stronger anti-inflammatory activity than their corresponding glycosides [36], where the glycosylation of some substituent groups may bring an important effect on the activities. The double bonds, especially at C-2/C-3 of ring C, in the flavonoid structure play a critical role in the anti-inflammatory activity [37,38], but not in isoflavone according to the results of research. Compounds **7**, **9**, **11**, **12**, and **13** showed similar NO inhibitory activities but different downregulated mRNA expressions of inflammatory factors; this functional difference may be related to the number and position of hydroxyl groups. Generally speaking, active compounds are the basic elements of TCM, as well as the main material base for disease treatment; thus, these compounds from SPC could be used as prototypes or lead compounds in the research and development of innovative drugs for treating inflammatory diseases, and further studies should be developed to evaluate the intracorporal process to reach the goal of elucidating the true material basis of their pharmacodynamics.

## Figures and Tables

**Table 1:** ^1^H (400 MHz) and ^13^C (100 MHz) NMR data for compounds **2** and **4** in dimethyl sulfoxide (DMSO)-*d*_6_.

No.	2		4	
	*δ*_H_ (*J* in Hz)	*δ*_C_ (mult.) ^a^	*δ*_H_ (*J* in Hz)	*δ*_C_ (mult.) ^a^
2	3.61, t (9.7) 4.29, dd (9.7, 3.5)	66.1 (CH_2_)	3.60, t (10.0) 4.22, dd (10.0, 3.7)	65.9 (CH_2_)
3	3.56, ddd (9.7, 7.0, 3.5)	39.4 (CH)	3.55, m	39.9 (CH)
4	5.52, d (7.0)	77.9 (CH)	5.50, d (6.8)	78.2 (CH)
5	6.99, d (8.5)	125.7 (CH)	7.24, d (8.4)	132.2 (CH)
6	6.55, d (8.5)	110.1 (CH)	6.47, dd (8.4, 2.4)	109.9 (CH)
7	–	151.3 (C)	–	158.9 (C)
8	–	135.8 (C)	6.27, d (2.4)	103.0 (CH)
9	–	149.7 (C)	–	156.5 (C)
10	–	112.5 (C)	–	111.4 (C)
1′	–	118.5 (C)	–	118.6 (C)
2′	–	153.9 (C)	–	153.9 (C)
3′	6.52, s	93.3 (CH)	6.52, s	93.4 (CH)
4′	–	147.6 (C)	–	147.6 (C)
5′	–	141.2 (C)	–	141.2 (C)
6′	6.97, s	105.5 (CH)	6.96, s	105.5 (CH)
3-OCH_3_	3.66, s	60.2 (CH_3_)	–	–
-OCH_2_O-	5.91, d (0.9); 5.95, d (0.9)	101.2 (CH_2_)	5.91, d (0.7); 5.94, d (0.7)	101.2 (CH_2_)

^a^ Attached protons determined by DEPT experiment.

**Table 2:** ^1^H (400 MHz) and ^13^C (100 MHz) NMR data for compounds **5** and **6** in DMSO-*d*_6_.

No.	5		No.	6	
	*δ*_H_ (*J* in Hz)	*δ*_C_ (mult.) ^a^		*δ*_H_ (*J* in Hz)	*δ*_C_ (mult.) ^a^
1	–	128.6 (C)	2	–	160.5 (C)
2	6.80, d (1.9)	113.2 (CH)	3	–	121.4 (C)
3	–	147.6 (C)	4	7.78, s	142.0 (CH)
4	–	145.3 (C)	5	7.24, s	109.7 (CH)
5	6.69, d (8.0)	115.5 (CH)	6	–	151.0 (C)
6	6.60, dd (8.0, 1.9)	121.1 (CH)	7	–	145.5 (C)
7	2.74, t (7.0)	36.7 (CH_2_)	8	6.81, s	102.6 (CH)
8	4.13, t (7.0)	65.0 (CH_2_)	9	–	149.1 (C)
1′	–	172.4 (C)	10	–	111.2 (C)
2′	2.44, t (6.7)	27.8 (CH_2_)	1′	–	120.6 (C)
3′	2.66, t (6.7)	34.1 (CH_2_)	2′	–	145.5 (C)
4′	–	209.1 (C)	3′	–	140.8 (C)
5′	2.39, t (7.4)	41.8 (CH_2_)	4′	–	151.8 (C)
6′	1.45, m	23.1 (CH2)	5′	6.64, d (8.6)	111.4 (CH)
7′	1.25, m	30.9 (CH_2_)	6′	6.86, d (8.6)	125.2 (CH)
8′	1.25, m	22.1 (CH_2_)	2′-OCH_3_	3.71, s	60.6 (CH_3_)
9′	0.85, t (7.1)	14.0 (CH_3_)	3′-OCH_3_	3.77, s	60.2 (CH_3_)
3-OCH_3_	3.74, s	55.7 (CH_3_)	4′-OCH_3_	3.82, s	56.2 (CH_3_)

^a^ Attached protons determined by DEPT experiment.

**Table 3:** ^1^H (400 MHz) and ^13^C (100 MHz) NMR data for compounds **14** and **15** in DMSO-*d*_6_.

No.	14		15	
	*δ*_H_ (*J* in Hz)	*δ*_C_ (mult.) ^a^	*δ*_H_ (*J* in Hz)	*δ*_C_ (mult.) ^a^
2	3.64, dd (9.8, 15.9)4.29, dd (9.2, 9.8)	66.1 (CH_2_)	3.92, t (10.2)4.12, br d (10.2)	69.6 (CH_2_)
3	3.66, ddd (6.4, 9.2, 15.9)	39.4 (CH)	3.35, m	30.9 (CH)
4	5.61, d (6.4)	77.9 (CH)	2.76, dd (15.6, 5.0)2.90, dd (15.6, 10.6)	30.0 (CH_2_)
5	7.39, d (8.6)	132.1 (CH)	6.87, d (8.5)	130.3 (CH)
6	6.72, dd (2.2, 8.6)	110.6 (CH)	6.29, dd (8.5, 2.4)	108.1 (CH)
7	–	158.6 (C)	–	156.6 (C)
8	6.56, d (2.2)	104.2 (CH)	6.19, d (2.4)	102.8 (CH)
9	–	156.4 (C)	–	154.9 (C)
10	–	114.3 (C)	–	113.1 (C)
1′	–	119.4 (C)	–	122.4 (C)
2′	–	160.4 (C)	–	156.3 (C)
3′	6.43, d (2.1)	96.5 (CH)	6.76, d (2.4)	102.0 (CH)
4′	–	160.7 (C)	–	159.2 (C)
5′	6.45, dd (2.1, 8.1)	106.3 (CH)	6.55, dd (8.6, 2.4)	107.3 (CH)
6′	7.26, d (8.1)	125.3 (CH)	7.07, d (8.6)	127.9 (CH)
1″	4.84, d (7.7)	100.5 (CH)	4.84, d (7.2)	101.3 (CH)
2″	3.22, m	73.3 (CH)	3.26, m	73.6 (CH)
3″	3.25, m	76.7 (CH)	3.27, m	77.0 (CH)
4″	3.14, m	69.8 (CH)	3.15, m	70.2 (CH)
5″	3.34, m	77.2 (CH)	3.35, m	77.5 (CH)
6″	3.67, m	60.8 (CH_2_)	3.73, m	61.1 (CH_2_)
	3.44, m		3.46, m	
4′-OCH_3_	3.70, s	55.4 (CH_3_)	3.72, s	55.3 (CH_3_)

^a^ Attached protons determined by DEPT experiment.

**Table 4:** The inhibitory activity of 15 compounds on nitric oxide (NO) production (*n* = 3).

Compounds	CC_50_ (μM)	IC_50_ (μM)	SI ^a^
1	26.23 ± 1.26	5.69 ± 0.28 ** ^▲▲▲^	4.61
2	>200 ^b^	46.26 ± 5.83	>4.32
3	>200	35.41 ± 2.92 ^▲^	>5.65
4	300.455 ± 11.98	40.05 ± 6.94 ^▲^	7.50
5	>200	45.87 ± 4.76	>4.36
6	702.91 ± 56.35	28.33 ± 6.44 ^▲^	24.81
7	335.09 ± 3.02	22.75 ± 0.55 ^▲▲^	14.73
8	314.68 ± 8.19	16.34 ± 4.45 ^▲▲▲^	19.25
9	3557.01 ± 925.86	16.87 ± 3.39 ^▲▲▲^	210.84
10	133.44 ± 2.11	6.78 ± 0.59 ** ^▲▲▲^	19.68
11	103.14 ± 9.56	21.11 ± 4.80 ^▲▲^	4.88
12	413.21 ± 3.075	35.49 ± 2.69 ^▲▲^	11.64
13	>200	36.43 ± 2.47 ^▲^	5.49
14	718.30 ± 110.98	122.56 ± 9.92	5.86
15	396.85	>200	<1.98
l-NIL	>200	19.08 ± 1.18	>10.48
IND	633.45 ± 75.12	55.44 ± 3.71	11.43

^a^ SI (Selectivity index) was calculated based on median cytotoxic concentration (CC_50_) half maximal inhibitory concentration (IC_50_). ^b^ CC_50_ could not be obtained because of a cell survival rate >98% at 200–6.25 μM. * *p* < 0.05, ** *p* < 0.01, *** *p* < 0.001 vs. l-*N*^6^-(1-iminoethyl)-lysine (l-NIL); ^▲^ *p* < 0.05, ^▲▲^ *p* < 0.01, ^▲▲▲^ *p* < 0.001 vs. indomethacin (IND).

**Table 5:** The sequences of the primers for qPCR.

Gene	Forward (5’–3’)	Reverse (5’–3’)
β-Actin	GGCTGTATTCCCCTCCATCG	CCAGTTGGTAACAATGCCATGT
IL-6	TAGTCCTTCCTACCCCAATTTCC	TTGGTCCTTAGCCACTCCTTC
IL-1β	TCAGGCAGGCAGTATCACTC	AGCTCATATGGGTCCGACAG
TNF-α	ATGGCCTCCCTCTCATCAGT	TGGTTTGCTACGACGTGGG
iNOS	TCCATGACTCCCAGCACA	CCATCTCCTGCATTTCTTCC
COX-2	GGCGCAGTTTATGTTGTCTGT	CAAGACAGATCATAAGCGAGGA
